# The Hallmarks of Glioblastoma: Heterogeneity, Intercellular Crosstalk and Molecular Signature of Invasiveness and Progression

**DOI:** 10.3390/biomedicines10040806

**Published:** 2022-03-30

**Authors:** Filippo Torrisi, Cristiana Alberghina, Simona D’Aprile, Anna M. Pavone, Lucia Longhitano, Sebastiano Giallongo, Daniele Tibullo, Michelino Di Rosa, Agata Zappalà, Francesco P. Cammarata, Giorgio Russo, Massimo Ippolito, Giacomo Cuttone, Giovanni Li Volti, Nunzio Vicario, Rosalba Parenti

**Affiliations:** 1 Section of Physiology, Department of Biomedical and Biotechnological Sciences, University of Catania, 95123 Catania, Italy; filippo.torrisi@unict.it (F.T.); cristiana.alberghina@phd.unict.it (C.A.); simonettadap@gmail.com (S.D.); am.pavone8@gmail.com (A.M.P.); azappala@unict.it (A.Z.); 2 Section of Biochemistry, Department of Biomedical and Biotechnological Sciences, University of Catania, 95123 Catania, Italy; lucialonghitano@hotmail.it (L.L.); sebastiano.giall@gmail.com (S.G.); d.tibullo@unict.it (D.T.); livolti@unict.it (G.L.V.); 3 Section of Anatomy, Department of Biomedical and Biotechnological Sciences, University of Catania, 95123 Catania, Italy; mdirosa@unict.it; 4 Institute of Molecular Bioimaging and Physiology, National Research Council—IBFM-CNR, 90015 Cefalù, Italy; francesco.cammarata@ibfm.cnr.it (F.P.C.); giorgio-russo@cnr.it (G.R.); 5 National Laboratory of South, National Institute for Nuclear Physics (LNS-INFN), 95125 Catania, Italy; cuttone@lns.infn.it; 6 Nuclear Medicine Department, AOE Cannizzaro, 95126 Catania, Italy; ippolitomas@yahoo.it

**Keywords:** glioblastoma, invasiveness, stress response, metabolism, immune modulation

## Abstract

In 2021 the World Health Organization published the fifth and latest version of the Central Nervous System tumors classification, which incorporates and summarizes a long list of updates from the Consortium to Inform Molecular and Practical Approaches to CNS Tumor Taxonomy work. Among the adult-type diffuse gliomas, glioblastoma represents most primary brain tumors in the neuro-oncology practice of adults. Despite massive efforts in the field of neuro-oncology diagnostics to ensure a proper taxonomy, the identification of glioblastoma-tumor subtypes is not accompanied by personalized therapies, and no improvements in terms of overall survival have been achieved so far, confirming the existence of open and unresolved issues. The aim of this review is to illustrate and elucidate the state of art regarding the foremost biological and molecular mechanisms that guide the beginning and the progression of this cancer, showing the salient features of tumor hallmarks in glioblastoma. Pathophysiology processes are discussed on molecular and cellular levels, highlighting the critical overlaps that are involved into the creation of a complex tumor microenvironment. The description of glioblastoma hallmarks shows how tumoral processes can be linked together, finding their involvement within distinct areas that are engaged for cancer-malignancy establishment and maintenance. The evidence presented provides the promising view that glioblastoma represents interconnected hallmarks that may led to a better understanding of tumor pathophysiology, therefore driving the development of new therapeutic strategies and approaches.

## 1. Introduction

In the era of personalized medicine, identifying and understanding pathophysiological mechanisms of cancer is a critical factor to shape therapies according to grade, histological features, molecular subtypes, aggressiveness, and response to treatment. To this regard, breast cancer therapy represents a prototypical model of treatment in clinical practice, personalizing the dosage and type of targeted drugs and radiotherapeutic treatment according to tumor phenotype and molecular classification (e.g., luminal A/B, HER+/−, basal-like, claudin-low) [1,2]. Such an approach contributes to an increase in the survival rate of patients and a decrease in adverse effects, allowing a better stratification of patients and clinical outcome [3,4]. Glioblastoma (GBM) represents an aggressive and invasive cancer, showing an incidence of 3.19/100,000 people per year and 15–18 months of median survival [5,6]. Unfortunately, the development of personalized therapeutic approaches for GBM are limited by its pathophysiological characteristics and its inter- and intratumor heterogeneity.

The first histological classification of nervous-system tumors provided by the World Health Organization has been revised several times in the last few decades by means of molecular data obtained from The Cancer Genome Atlas omics and other complementary information coming from genomic, proteomic, and metabolomic studies [7,8,9]. Despite this effort, genetic and molecular subtypes of nervous-system tumors are treated using similar approaches based on the Stupp regimen, approved in 2005 and amended in the 2017 by adding tumor-treating fields. The current treatment for newly diagnosed GBM is not an exception to this standard approach and is based on surgery, if possible, followed by the alkylating agent temozolomide (TMZ) used in combination with 60 Gy of X-ray irradiation, fractionated in 30 sessions of 2 Gy each, with minor modifications in case of treating-field addition or hypofractionation in elderly patients [10,11]. Additional therapeutic approaches can be grouped into three categories: chemotherapy drugs, molecularly targeted drugs, and immunotherapies. Major chemotherapy drugs include nitrosoureas for their good blood–brain-barrier (BBB) penetration and better efficacy in patients with O6-methylguanine—DNA methyltransferase (MGMT)-methylated tumors [12]; there are no data suggesting the benefits of other chemotherapeutic agents such as carboplatin, procarbazine, irinotecan, and etoposide, which are mostly used for recurrent GBM [13]. Targeted therapies include agents blocking both downstream and upstream-signaling pathways by acting on growth factor receptors, ligands, and second messengers of cell-signal transduction [14]. Immunotherapies can be considered among the targeted therapies, especially for the inhibitors of the main immunosuppression molecules such as transforming growth factor beta (TGF-β) or colony-stimulating factor. Current direct immunotherapies can be summarized in three subsets: vaccines; checkpoint inhibitors/monoclonal antibodies; virotherapies and chimeric antigen receptor T (CAR T) cells. Challenges are focusing on multiple-antigen recognition and on intrinsic/adaptive resistance overcoming the so called immunologically “cold” microenvironment of GBM, to promote a robust antitumor immune-cell response [15].

Therefore, an up-to-date knowledge of cancer hallmarks driving GBM initiation, maintenance, and progression becomes crucial to overcome the intrinsic mosaicism and to meet the clinical need for personalized, targeted, and effective therapies. Recently, many efforts have been directed to identify key factors inducing GBM tumorigenesis and progression. In particular, research in the field has been focused on homocellular and heterocellular interactions between cancer cells, cancer stem cells, fibroblasts, nervous cells, endothelial cells, blood vessels, and the extracellular matrix (ECM) [16]. These interactions promote the formation of a complex tumor microenvironment (TME), where cancer hallmarks acquire a holistic connotation, characterized by the crosstalk among cell populations influencing and supporting each other.

In this review we aim to analyze the hallmarks of GBM, highlighting their mutual crosstalk, impacting the complex tumor network. Firstly, we describe the main GBM hallmarks following Fouad and Aenei’s definition of “cancer hallmarks” [17]. This definition updated the one described by Hanahan D. and Weinberg R.A. [18,19], which has been improved taking advantage of genetic, molecular, environmental, and phenotypic information of recent research [17].

**Figure 1:**
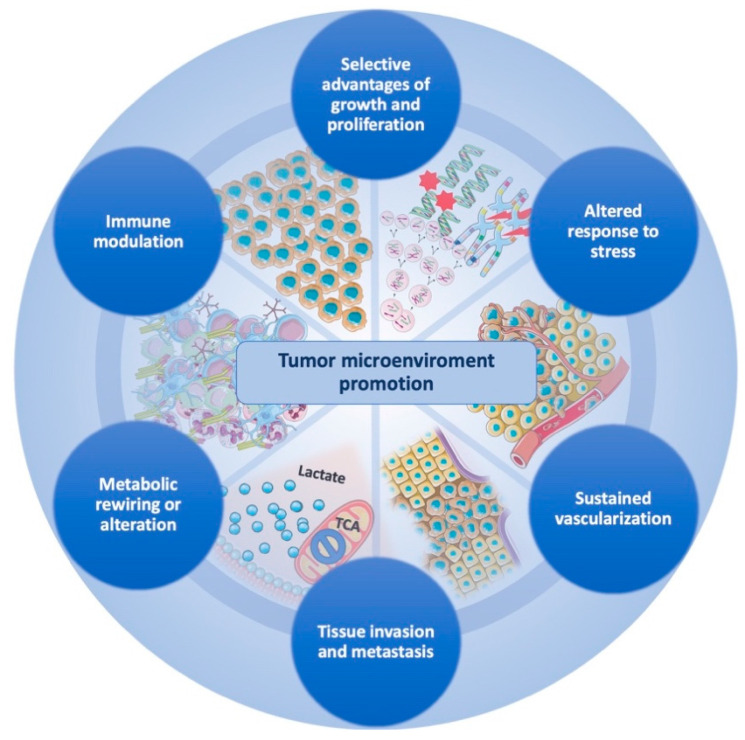
Glioblastoma-tumor microenvironment promotion is ensured by the coexistence of interconnected cancer hallmarks that act with several strategies to achieve tumor maintenance and progression. The circular structure holding the hallmarks emphasizes the cooperation that GBM cells exert to create an ultimate tumor microenvironment that supports survival and progression of cancer cells.

The newly proposed cancer hallmarks fit well to describe GBM, where the potential overlap between biological processes is even more evident than in other cancer diseases. In particular, GBM stem cells (GSCs) or, more generally, glioma/tumor-initiating cells, are characterized by these hallmarks and are involved in GBM pathogenesis and progression, according to the glioma stem-cell-hypothesis model [17,20]. On this basis, the present review analyzes the following seven hallmarks of GBM: (1) selective advantages of growth and proliferation; (2) altered response to stress; (3) sustained vascularization; (4) tissue invasion and metastasis; (5) metabolic rewiring or alteration; (6) immune modulation; (7) TME promotion, to be considered as a combination of all previous (Figure 1).

## 2. Selective Advantages of Growth and Proliferation

**Figure 2:**
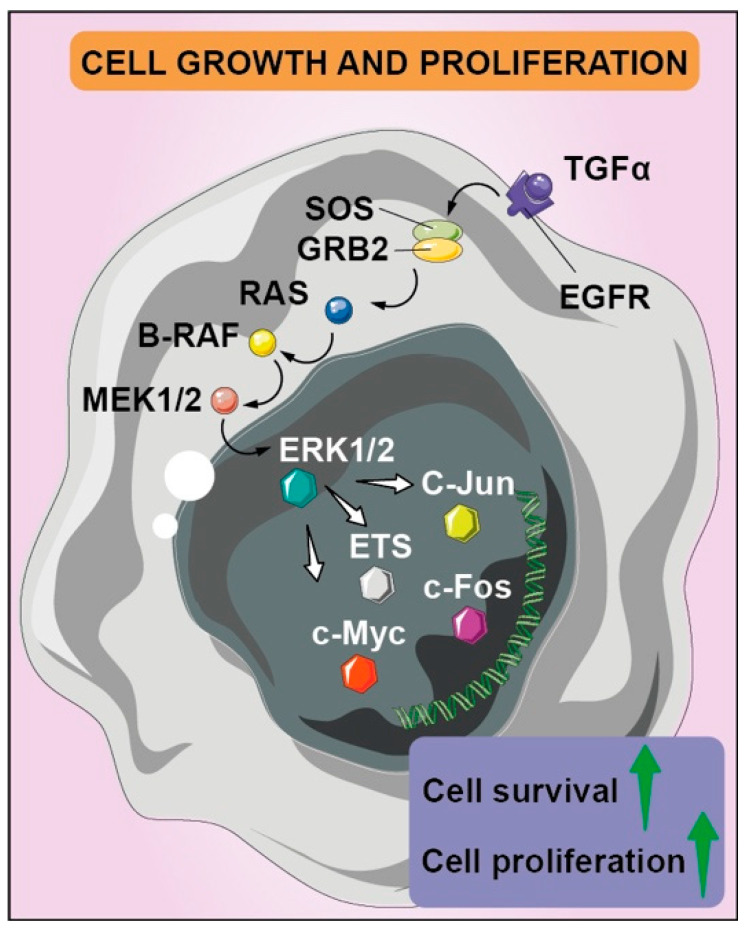
Aberrant regulation of tyrosine kinase receptors determines several signaling pathways’ activation, including mitogen-activated protein kinase cascades with pleiotropic effects, such as cell proliferation and survival. EGFR and its mutated version EGFRvIII’s overexpression and/or hyper-activation is also fostered by autocrine feedback loop that leads to the transcription of EGFR ligands such as TGFα; the downstream effector Grb2 activates guanine nucleotide exchange factor SOS for GDP/GTP cycling, which promotes formation of active Ras-GTP, binding the other downstream effector targets such as B-Raf. It phosphorylates MEK1 and MEK2 dual-specificity protein kinases, which determine the ERK1 and ERK2 nuclear translocation, where they find several protein targets, such as transcription factors of Ets family, c-MYC and c-JUN. The latter forms a heterodimer with c-FOS, leading to increased cell survival, proliferation, and invasion.

The main hallmark of malignant neoplasms is the uncontrolled growth and proliferation index as a result of genes and signaling-pathway deregulation. The activation of oncogenes and inactivation of tumor-suppressor genes, with the contribution of epigenetic factors, are involved in several interconnected pathways [21], so that intracellular signaling appears unbalanced, inducing cell growth and proliferation, but also contributing to additional hallmarks of cancer. The Ras-Raf-MEK-ERK pathway, one of the main mitogen-activated protein kinase (MAPK) pathways, certainly stands out for its overall biological significance, modulating a number of either cytosolic and nuclear proteins involved in cell proliferation, survival, and metastasis (Figure 2). The activation of the Ras-Raf-MEK-ERK pathway supports the growth and proliferation potential—both with direct effects, such as alteration of the cell cycle, and indirect effects, such as the creation of a favorable TME and new vascularization, which guarantees the availability of nutrients and growth factors [22].

High-throughput approaches revealed converging signaling pathways with a number of common nodes, determining downstream effects on promising therapeutic targets [23,24,25]. In particular, most of the receptor tyrosine kinases (RTKs) are involved, including VEGFR, PDGFR, c-MET, EGFR and its mutated cognate EGFRvIII [23]. The SRC family of protein tyrosine kinases is also holding great promises for GBM therapy [26]. Moreover, the upstream stimulation of RTKs in turn triggers the activation of SRC proteins, not only promoting uncontrolled growth processes, but also vascularization, migration, invasion, and cell survival [27,28]. Finally, downstream effects in the nucleus include growth-related protein transcription and death-related factor inactivation. Consequently, a number of preclinical and clinical studies revealed that GBM can be induced and maintained by aberrant EGFR and Ras/RAF/ERK-signaling networks, also capable of revealing important downstream factors including Akt or mTORC1 signaling, associated with a benefit from the EGFR-targeting antibody nimotuzumab [29]. Furthermore, in vivo studies strongly suggest that suppression of Ras signaling is sufficient to suppress the tumorigenic potential of the glial progenitor cells [30,31]. In addition, the Ras-RAF-ERK pathway is also involved in the metabolic rewiring of GBM, caused by the deregulation of pyruvate dehydrogenase phosphatase, eventually inhibiting pyruvate dehydrogenase activity and leading to an attenuated mitochondrial reserve capacity [32].

A lot of evidence has come to suggest that deregulation of the p16(INK4a)-Cdk4/6-Rb axis correlates with GBM. Multiple phosphorylation events of tumor-suppressor retinoblastoma protein (RB) facilitate progression into the S-phase, unlocking the cell cycle at the G1/S checkpoint [33]. The majority of GBM patients indeed show CDKN2A, CDK4, or RB gene alterations, with consequential deregulation of the p16-cdk4-pRb cell-cycle regulatory cascade [34,35]. The RB pathway, in its inactivated state, drives GBM apoptosis evasion and confers resistance to DNA damages, also stimulating autophagy as a response to stress [36]. RB1-negative regulation was also found to induce cell-cycle regulators (i.e., N-myc) overexpression in GSCs, under the constitutive activation of Sonic hedgehog (SHH), due to the hereditary loss of function mutations in its receptor, Patched [37]. Evidence coming from double-knockout mice for p16Ink4a/p19ARF reported an upregulation of Bmi1, a promoter of neural stem-cell self-renewal [37]. It is worth noticing that not only growth factors but also other stimuli may interact with RB1 pathways. such as a plethora of inflammatory molecules; consistently, reactive oxygen species (ROS) and nitrogen species are common inflammatory mediators participating in the GBM neoplastic process, with other inflammatory molecules, which phosphorylate RB1 leads to its inactivation [38].

In physiological conditions, the p53-ARF-MDM2 pathway controls genomic integrity, activating cell-cycle checkpoint genes, mediating cell-cycle arrest and/or apoptosis induction. MDM2, a ubiquitin ligase, promotes p53 degradation, modulating its activity by a negative feedback loop. Among the modulators, ARF blocks p53 degradation, preventing MDM2 translocation out of the nucleolus [39]. The p53-ARF-MDM2 axis is one of the most frequently mutated pathways in GBM, accounting for 84% of patients and 94% of GBM cell lines with a major prevalence for the proneural and mesenchymal subtype [40]. Hyperproliferation, absence of apoptotic response, and invasive pattern are processes correlated with p53 deregulation, promoting cancer progression in response to DNA damage, genotoxicity, oncogene activation, and aberrant growth signals [40].

These aforementioned oncogenic processes for the selective advantages of growth and proliferation are well-exploited by GSCs, representing the main drivers for tumor progression thanks to their sustained self-renewal and persistent proliferation [41]. Multiple factors are implied in the maintenance of stemness, such as their interaction with TME components, vascular compartment, mesenchymal stem cells, and immunity [42]. Indeed, GSCs increase their survival and maintenance, activating typical stem-cell developmental programs, such as aberrant Notch, NF-κB, PDGFRβ, EGFR, and TGF-β signaling [41]. EGFR, mTOR, PI3K, MET, SRC represent interesting targets for selective antibodies or enzymatic inhibitors for GBM therapy. Among these, antibody-drug conjugate Depatuxizumab Mafodotin to inhibit EGFR has been tested, either as a single treatment or in combination with temozolomide [43]. Everolimus and Temsirolimus were tested against mTOR. The latter was associated with increased effects on tumors with phosphorylation of mTOR Ser2448, identifying a subgroup of patients that may benefit from mTOR inhibition [44]. Buparlisib was tested in patients with recurrent GBM to inhibit PI3K, with a significant brain penetration, but a minimal single-agent efficacy and incomplete PI3K-pathway inhibition [45].

## 3. Altered Response to Stress

GBM cells can preserve their survival and progression potential thanks to the control of an efficient DNA damage/repair system. MGMT is a key enzyme that confers this property, removing alkylating agents from DNA, and protects against the effect of chemotherapeutic agents such as TMZ [46]. For this reason, epigenetic changes, such as the inactivation of MGMT by hypermethylation of its promoter, may represent a robust positive predictive and prognostic biomarker for patients associated with better therapeutic efficacy and longer life expectancy [47,48]. Epigenetic changes and genomic instability are related to the alteration of GBM metabolism: the mutation status of isocitrate dehydrogenases (IDHs) is responsible for enzymatic modifications, triggering the Glioma CpG Island Methylation phenotype (G-CIMP), the main factor inducing epigenetic changes [49]. In GBM cells, the main forms of IDH are heterodimers containing wild-type IDH1 and R132H mutation monomers, exhibiting neomorphic activity that reduces α-ketoglutarate (α-KG) into D-2-hydroxyglutarate (D-2-HG) with NAPDH consumption and NADP+ production. The generation of D-2-HG inhibits demethylation by reducing α-KG-dependent-dixoxygenase function on Ten-eleven translocation methyl cytosine dioxygenase, which catalyzes the cytosine demethylation steps. The D-2-HG-mediated demethylation decrease is also promoted by the lysine-specific demethylase inhibition [50]. Nevertheless, the IDH wild-type GBMs are protected from oxidative damages and ROS due to the regeneration of reduced glutathione by cytosolic and mitochondrial NADPH production, which is consumed in IDH-mutated GBMs [51,52]. Therefore, IDH wild-type GBMs are more aggressive as compared to IDH-mutated GBMs; the latter is usually a secondary GBM and/or a low-grade glioma, such as oligodendroglioma and pediatric glioma, associated with a better prognosis [53]. However, it should be highlighted that there is an additional mechanism related to the higher overall survival of IDH-mutated GBM patients. This is linked to ATP synthesis reduction in tumor cells, through the conversion of α-KG to a lower-energy substrate (i.e., D-2-HG), mediated by IDH1- and IDH2-mutated isoforms [54]. Summarizing, the correlation between aggressiveness and IDH status supports the consideration that epigenetic changes and metabolic rewiring cooperates for GBM initiation or progression. In addition, it has been observed that DNA single-strand breaks and double-strand breaks induced by chemotherapeutic agents or radiation treatment are bypassed by efficient repairing mechanisms, and sensor and effector molecules that are collectively indicated as DNA damage response (DDR) [55,56]. DDR in GBM is particularly active both for single-strand and double-strand break repair; GSCs retain a constitutive activation of several components of DDR, such as with DNA-PK, ATM, ATR, and cell-cycle checkpoint pathways [57].

ROS can be considered a critical indicator of cellular homeostasis, strictly dependent on the correct balance of their production and removal. Cell death caused by cellular oxidative damage occurs when damage is not reversible anymore. However, as in the case of such a highly resistant tumor, tumor cells adapt and actually increase their invasiveness. Hypoxic TME sustain ROS detoxification by HIF1α stabilization and by superoxide removal controlled by lactate [58].

**Figure 3:**
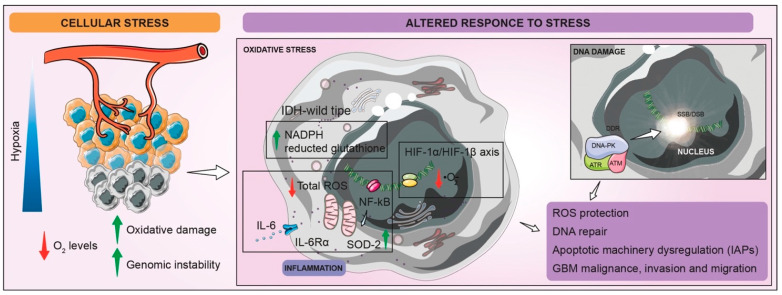
Schematic representation of altered GBM cells responses to stress. The typical condition of hypoxia that characterize GBM TME leads to an increased amount of mitochondrial and intracellular ROS and heightened genome instability. Low levels of O_2_ induce stabilization and activation of HIF-1α/HIF-1β axis, which improves superoxide-anion (·O_2_^−^) removal. Inflammatory state of hypoxic cells allows total ROS detoxification by IL-6/IL-6Rα enhancing and mitochondrial SOD-2 production, guided by NF-κB signaling-pathway activation. Moreover, restored levels of reduced glutathione by cytosolic and mitochondrial NADPH production in IDH wild-type GBM guarantee the protection from oxidative stress. Finally, stress condition also stimulates DDR, involving DNA-PK, ATR, and ATM recruitment, which ameliorates SSB/DSB repair system. All of these processes aim to overcome oxidative stress and DNA instability, ensuring increased GBM malignance, invasion, and migration.

A strong indication of an ongoing metabolic reprogramming contributing to resistance is mitochondrial oxygen-consumption-rate reduction, caused by pyruvate dehydrogenase kinase 1 inhibition, hence decreasing the oxidation of pyruvate in mitochondria, and increasing the conversion of pyruvate to lactate [58]. ROS signaling is strongly interconnected to the inflammation hallmark in GBM. IL-6 production, a prosurvival factor for GBM, inducing a signal transducer and activator of transcription-3 (STAT-3) activation in GSCs, plays a key role in the inhibition of intracellular and mitochondrial ROS. Moreover, STAT-3 is involved in the interplay between extracellular signals and transcriptional pathways, leading to proliferation and cell-cycle progression. NF-κB-signaling pathways have a prominent role in proinflammatory-molecule production, including IL-6, driving not only cell proliferation but also protection from ROS by the positive regulation of some antioxidant enzymes, such as mitochondrial SOD-2 (Figure 3) [58].

Dysregulation of the apoptotic machinery is certainly a focal point of an altered response to stress, promoting tumor development [59,60]. In GBM, intrinsic and extrinsic apoptotic mechanisms are altered by pathway activation controlling growth and proliferation determining an aberrant response to stress, such as oxidative stress and hypoxia. PI3K/AKT/mTOR signaling, via PI3K-mediated Akt phosphorylation, induces phosphorylation of many downstream targets, inducing NFκB activation, mTORC2, and MDM2 [61]. The final effect is the activation of inhibitor of apoptosis proteins (IAPs) inducing caspase-3, caspase-7, and caspase-9 inhibition and the downregulation of p53. STAT-3, the Notch signaling pathway and SHH pathway were also associated to the control of apoptosis and proliferation [62,63,64]. It is also recognized that the antiapoptotic protein Bcl-2 family’s overexpression in GBM-cell lines stimulates migration and tumoral invasion.

Hypoxia is strictly interconnected to the aberrant response to stress. In particular, during the transition from the first vascular phase, in which cancer cells are supported by native blood vessels, to the neovascularization phase, a vascular-cell apoptotic program activated by Ang-2 expression in GBM cells, followed by vascular involution and finally to downstream effects stimulating the neoangiogenesis by VEGFR transcription [65,66]. Hypoxic stress turns out to be a key strategy that GBM adopts to support its progression by HIF-1α/HIF-1β axis activation, targeting many effector genes and driving several interconnected hallmarks. It is also worth noticing that autophagy, the main catabolic process complementary to the ubiquitin–proteasome system, is activated when cells are exposed to various types of stress, such as oxygen deprivation and nutrient starvation [67]. The induction of autophagy in hypoxic conditions is mediated by the activation of several autophagy-related proteins, such as REDD1 and BNIP3, which relieve mTOR inhibition, thus decreasing the ATG1/ULK1 complex phosphorylation that initiates autophagy [67]. In addition, hypoxia stimulates adenosine monophosphate-activated protein kinase (AMPK), which not only coordinates tumor bioenergetics and glycolysis in GBM, but also phosphorylates TSC2 and ULK1, activating both mTOR dependent and independent autophagy [67,68]. The importance of the autophagy mechanism on GBM leads to the investigation of gene-expression profiles and clinical data in order to find the prognostic value of autophagy-related genes; differentially expressed autophagy-related genes were found in lower-grade gliomas and in relation with TME [69,70]. DNA-damaging agents represent the most effective strategy to impair the DDR system. Velaparib and Olaparib are the most common DDR inhibitors in clinical phase, but ATM, DNA-PK and Wee1 are also promising targets to increase unrepaired double-strand breaks. Current studies are aiming at identifying predictive biomarkers of sensitivity to specific DDR inhibitors [71].

## 4. Sustained Vascularization

Despite the large presence of necrotic and hypoxic areas, GBM is considered a highly vascularized tumor, and the sustained vascularization is a critical mechanism supporting GBM progression and growth [72]. Firstly, the vascularization of GBM is not only related to the angiogenic process triggered by the main axis of hypoxia/hypoxia response element (HRE) sequence transcription/VEGF activation, but also by many types of vessel-generation processes, which can be distinguished into five types: (i) angiogenesis; (ii) vasculogenesis; (iii) vascular co-option; (iv) vascular mimicry; and (v) glioblastoma-endothelial-cell transdifferentiation [73]. The correct chronological sequence for the progression of these processes is still not clear, but the involvement of genes such as VEGF, erythropoietin, platelet/endothelial-cell-adhesion molecule 1, matrix metalloproteinases, and inhibitor of metallopeptidase, are common features [74]. Although angiogenesis is the most prevalent term used to indicate vascularization, it is not the first phenomenon guiding vessel formation, because the vascular co-option, with the overexpression of angiopoietin-2, begins the vascularization process around normal microvessels in GBM, induced by both hypoxic and nonhypoxic stimuli. Vasculogenesis is mainly driven by bone-marrow-derived cells, whereas vascular mimicry and GBM-endothelial-cell transdifferentiation are associated to the maintenance of a stem-cell phenotype [73]. The relevance of vascularization processes in GBM is the rationale of anti-VEGF therapies (i.e., Bevacizumab), but the absence of significant improvements on the mean overall survival reduced the enthusiasm of its use in the clinical practice for GBM [75]. The failure of bevacizumab may be considered an example indicating the existence of additional, compensative, and integrative mechanisms that sustain vascularization in GBM. Indeed, it has been reported that GBM cells can react against anti-VEGF therapy, promoting additional hallmarks of cancer. Huveldt et al. showed that bevacizumab induced tumor invasion and metastasis by the activation of SRC, which is a key factor of multiple pathways in cancer progression [76]. Metabolic reprogramming is also adopted in response to antiangiogenic therapy. Kuang et al. showed that bevacizumab-resistant cells upregulated glucose transporter 3, establishing a Warburg effect-mediated resistance mechanism [77]. Acidification in TME stimulates VEGF and fibroblast growth factor β expression, accelerating the proliferation and motility of endothelial cells and the budding of the vascular system through monocarboxylate transporter-4 (MCT-4)-mediated lactate release, which promotes the production of hyaluronic acid, strongly supporting vessel formation [78].

**Figure 4:**
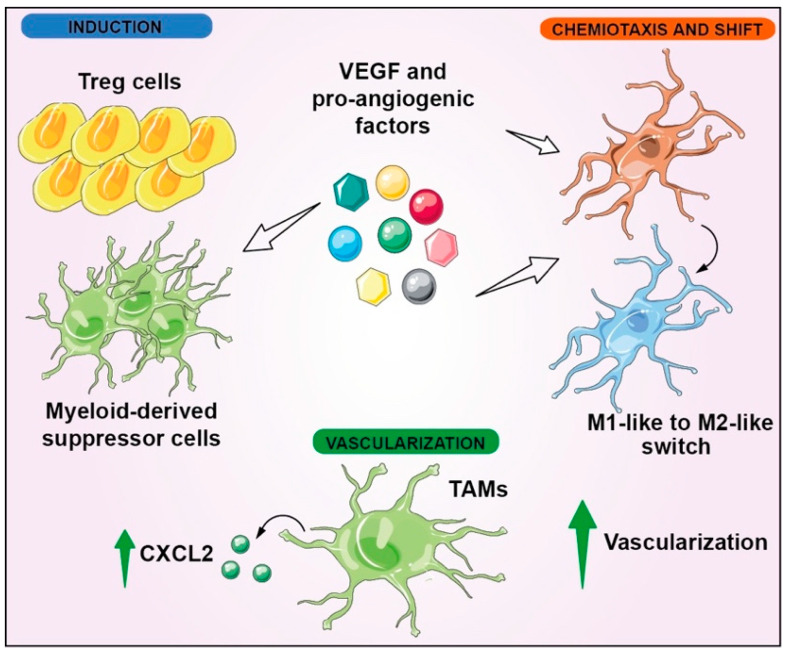
Vascularization and immunomodulation interconnection in GBM. VEGF induces Treg cells and MDSCs; furthermore, it stimulates M1-like to M2-like switch in microglial cells, leading to immune evasion. Moreover, proangiogenic factors, including VEGF, induce chemiotaxis of microglial cells. Instead, resident TAMs promote vascularization, upregulating the cell-signaling cytokine CXCL2.

Since the formation of new vessels is involved in enhanced inflammation and vice versa, sustained vascularization and immunomodulation can be considered two interconnected hallmarks in GBM. In this scenario, TAMs have a key role in promoting vascularization, increasing the expression of hematopoietic markers such as CD45, and upregulating CXCL2 (Figure 4) [79,80,81]. The reciprocal communication between dendritic cells (DCs) and vascularization has been verified by the observation that the proangiogenetic factors were able to induce chemotaxis and an M1-like to M2-like phenotype switch in microglial cells (Figure 4) [82].

A correlation between vascularization and immunomodulation has also been reported by Souberan et al., who described the involvement of monocytes and DCs in tumor immune reprogramming as a resistance mechanism upon anti-VEGF therapy [83]. Blood-vessel development in GBM is also regulated by RAS/RAF/ERK signaling [84], determining a positive feedback loop with VEGFR. Even if RTKs are commonly considered upstream activators of the signal-transduction mechanisms, it is worth noticing that the activation of Ras-RAF-ERK by RTKs can determine the transcription of HIF1α, which in turn promotes the transcription of VEGF, therefore increasing VEGFR activity [85]. Immunomodulatory mechanisms are also triggered by angiogenesis, because regulatory T cells (Treg cells) and myeloid-derived suppressor cells (MDSCs) are induced by VEGF, which also promotes an M2-like phenotype shift in macrophages exhibiting immunosuppression functions (Figure 4) [86].

## 5. Tissue Invasion and Metastasis

**Figure 5:**
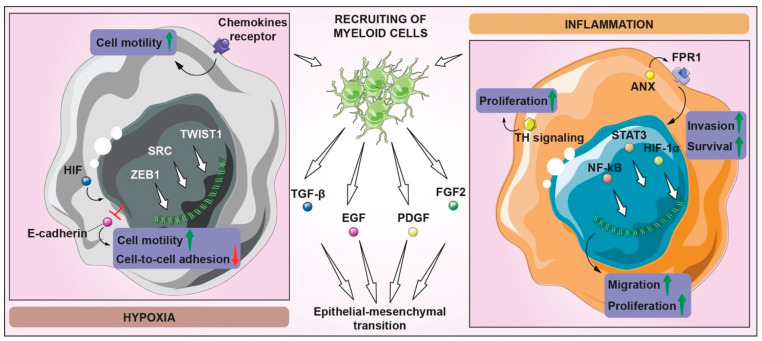
Tissue invasion and metastasis in GBM. Many pathways are involved in the invasion processes of GBM, especially in hypoxic and inflammatory contexts. Hypoxic and inflammatory conditions lead to the recruitment of myeloid cells, which promote TGF-β, EGF, PDGF, and FGF2 signaling, triggering the EMT initiation. In hypoxic conditions, HIFs regulate ZEB1, inhibiting E-cadherin expression, which contributes to the loss of cell–cell adhesion and the increase in motility, also supported by the activation of TWIST1, SRC, and chemokine receptors. In the inflammatory context, the upregulation of proinflammatory genes determines an increase in migration and proliferation, while ANX, through the FPR1 activation, induces the heightening of invasion and survival ability by means of STAT3, NF-kB, and HIF-1α pathways. Moreover, proliferation is also promoted by TH signaling, derived from microglia-GBM crosstalk.

Migration and motility are essential cell functions allowing cancer cells to migrate from the primary site to nearby or distant sites. GBM is characterized by local infiltration rather than the spreading beyond the central nervous system (CNS), even if this characteristic is enough to strongly compromise physiological functions and quality of life of patients. Although the spread of metastasis outside the CNS is rare, the invasion ability of GBM through the healthy parenchyma and stroma is the main factor in inducing resistance and recurrences [87]. Such a GBM feature is a clear example of the difference between invasiveness and metastasis. Two factors may explain the infrequent GBM migration and growth in secondary organs: the first is based on or due to the relatively short mean overall survival of patients; the second is related to the physical barriers of the CNS skull and BBB [88]. However, Lah T.T. et al. demonstrated that even when the BBB blocks circulating tumor cells, the peculiar molecular and genetical features of GSCs drive infiltrative potential during early tumor growth, both locally and to distant niches. GSCs residing in a protective vascular-invasive niche are able to migrate and invade deeply into the brain parenchyma, also supporting a microenvironment for survival, growth, and immune surveillance [89]. Many of the TME features are linked to GBM invasiveness; in fact, proteases and transcription factors required for the initiation of epithelial mesenchymal transition (EMT), are triggered by TGF-β, EGF, PDGF, and FGF2, produced by circulating or residential myeloid cells, which are recruited in hypoxic and inflammatory conditions [90]. In the hypoxic condition, HIFs regulate the zinc-finger E-box-binding homeobox ZEB1, which suppresses E-cadherin, increasing cell motility and losing cell-to-cell adhesion [90]. However, a plethora of events correlate hypoxic TME GBM invasion and are linked with the remodeling of the cytoskeleton, ECM degradation, and motility under the control of key signaling pathways such as SRC, TWIST1, and chemokine receptors [91,92,93,94]. In hypoxic pseudopalisading GBM cells, proinflammatory-gene expressions, such as C-X-C chemokine receptor type 4 (CXCR4), are upregulated by HERs-gene transcription, stimulating migration but also the proliferation of endothelial cells close to necrotic or hypoxic areas [95]. Inflammation, proliferation, and invasion interplay have been also related to the G-protein-coupled chemoattractant receptor, formylpeptide receptor 1 [96], which is activated by Anx releasing from tumor necrotic cells, determining cell migration, growth, and production of angiogenic factors mediating the SRC-EGFR signaling pathway, leading to ERK/F-actin axis activation and cell chemotaxis. This sustains the invasion and survival through the phosphorylation of transcription factors NF-κB, STAT3, and HIF-1α [96]. Inflammatory stimuli inhibit tumor invasion through TLR/MAPK signaling, increasing proliferation by TH and STAT3-signaling activation (Figure 5) [97].

The intricate network, which positively and negatively regulates GBM invasiveness, has been extensively studied to develop therapeutic approaches, including the PI3K/Akt, Wnt, SHH-GLI1, and microRNAs [98].

## 6. Metabolic Rewiring and Adaptation

**Figure 6:**
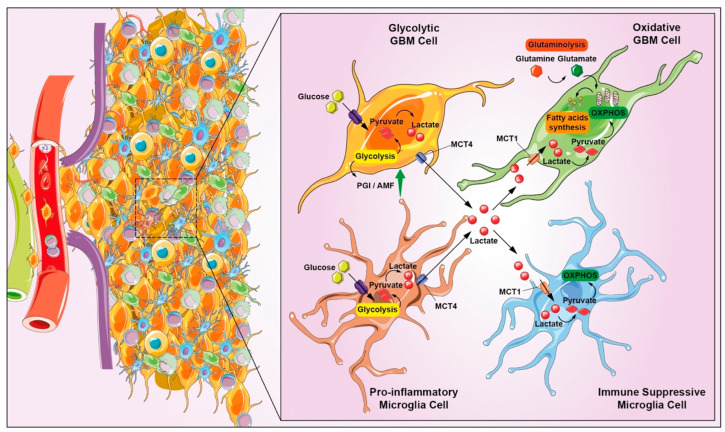
Representation of metabolic reprogramming processes connected GBM invasiveness. The support of GBM invasiveness in hypoxic conditions is not only directly linked to lactate production, but also by indirect effects such as the production of additional signals of molecules such as PGI/AMF, and lipid metabolism and amino-acid reprogramming offers an important contribution to the proinvasion phenotype. Microglia adopts aerobic glycolysis by increasing lactate production and promoting M2 polarization with concomitant production of cytokines and other factors that can directly suppress effector cells, or indirectly via other types of immune cells such as intratumoral DCs and Treg cells, resulting in immunosuppression.

The ability of GBM to invade adjacent tissue is enhanced by metabolic reshaping, providing energy and adapting to survive in a complex and hostile environment, characterized by reduced nutrient and oxygen availability. The invasiveness support of GBM in hypoxic conditions is not only directly linked to lactate production, increased acidification rate, and expression of specific enzymes, but also by indirect effects mediated by the production of additional signaling molecules synergistically acting with enzymes, such as phosphoglucose isomerase/autocrine motility factor (PGI/AMF) [99]. PGI/AMF is an autocrine-motility factor, stimulating cell migration in vitro and metastasis in vivo [100]. Not only glycolysis, but also lipid, amino-acid, and nucleotide-metabolism reprogramming offers an important contribution to sustain invasiveness of GBM (Figure 6). Higher fatty-acid (FA) synthesis promotes its FA-uptake channel CD36 upregulation, associated with a proinvasive phenotype. Regarding amino-acid metabolism, glutamine and arginine have been associated to cell-adhesion mechanisms and nitrogenous-base accumulation, which are required for tumor invasion [101].

Hence, the evidence that several metabolic participants take part in GBM invasiveness sustainment confirms that the metabolic modulation cannot be summarized just referring to the Warburg effect, especially for such a highly heterogeneous tumor as GBM. Indeed, glycolysis, FA oxidation, and glutaminolysis were found differently involved in the metabolic reshaping in primary, immortalized, and patient-derived GBM cells, together with a multitude of dysregulated bioenergetic pathways, coexisting within the tumor and concomitant glycolysis or oxidative-phosphorylation-driven metabolism in subpopulations of GBM cells [54,102,103]. The opposite shift, from glycolytic to oxidative phosphorylation, has been described as a reverse Warburg effect in many cancer types, including GBM, suggesting adaptive mechanisms of cells according to TME modulation [104,105]. In addition to the typical enhanced aerobic glycolysis of cancer cells, the GBM glycolytic process is also supported by hypoxic TME, in which HIF-1α ubiquitination is limited by prolyl hydroxylase inactivation. This process induces HIF-1α/HIF-1β heterodimer stabilization, binding the HRE sequence and allowing the transcription of several enzymes, such as glycolytic enzymes, glucose transporters, and lactate dehydrogenase A, increasing GBM cells’ adaptation to hypoxic conditions [106]. The first event inducing a metabolic rewiring was the decrease of intracellular pH, due to the accumulation of lactate and protons, which give rise to other hallmarks. The oncometabolite function of lactate is also associated with the immunosuppressive environment constitution; indeed, lactate promotes an M2-like immunosuppressive macrophage polarization [107]. The link between metabolic rewiring and immunomodulation can be considered a chief interconnected hallmark. Direct evidence about this correlation has been reported, such as the role of natural killer group 2 member D (NKG2D) on NK cells via induction of NKG2D ligands on myeloid cells that are downregulated by LDH isoform 5 released from GBM cells and detectable in blood serum [108]. Moreover, LDHA inhibition by diclofenac has been related to IL-2 production and Toll-like receptor stimulation, inducing immunosuppression in a GBM preclinical models [109]. Oxidative stress and reduced oxidative phosphorylation induce ROS production and proinflammatory response in microglia; especially in hypoxic niches, microglia adopt the aerobic glycolysis, increasing lactate production and promoting an M2-like polarization, stabilizing HIF, further contributing to the immunosuppressive mechanisms (Figure 6) [110]. In addition, 2-HG derived from the activity of mutated IDH1/IDH2, inhibits microglial activation via the AMPK/mTOR/NF-κB pathway, limiting the inflammatory responses [111]. Moreover, as per microglial cells, reactive astrocytes also participate in the complex metabolic reprogramming via JAK/STAT-pathway activation supported by microglia-derived factors [112]. Reactive astrocytes respond to hypoxic-condition-enhancing alternative sources of glucose via gluconeogenesis or ketolysis and mediate a recruitment of M2-like TAMs [110]. MDSCs are considered the main immune components involved in the metabolic crosstalk between tumor cells and the immune system; immunosuppression takes place by the metabolic reprogramming of MSDCs via Arg1, iNOS, and indoleamine 2,3 dioxygenase 1 (IDO1) inhibition, which determine amino-acid depletion and CD8+ and natural killer cells’ suppression [113].

Preclinical evidence suggesting the efficacy of metabolic reprogramming and of oncometabolite-targeting drugs is increasing overtime. To date, Ivosidenib, an IDH inhibitor, has been tested in Phase I and results are supporting further research targeting it [114]. Other ideal targeting genes regulating tumor metabolism may be represented by PTEN-induced kinase 1 (PINK1)243 and hexokinase 2 (HK2). Indeed, inhibition of HK2 by ketoconazole and posaconazole revealed an inhibitory effect on GBM both in vitro and in vivo [115]; activation of PINK1 suppresses ROS and tumor growth through FOXO3a and reduces in vivo glioblastoma growth in orthotopic mouse xenograft models [116]. However, a plethora of RTK-driven metabolic dependencies have also been identified, and these include EGFR amplification for glucose uptake, glycolysis, NAD^+^ production, cholesterol uptake, fatty-acid (FA) synthesis, epigenetic remodeling, and membrane lipid. Metformin, Gboxin, and IACS-010759 are the main oxidative phosphorylation (OXPHOS) inhibitors blocking the electron transport chain [117].

## 7. Immune Modulation

Despite BBB being responsible for creating an immune-privileged microenvironment in the CNS and in brain tumors (known as cold TME), several immune-cell types have been recently associated with GBM progression. Tight junctions of endothelial cells are weakened under inflammatory conditions in GBM, changing BBB permeability and selectivity [118]. Bone-marrow-derived macrophages, MDSCs, microglia, DCs, and neutrophils are the main component of immune cells in GBM [119]. It has been reported that GBM subtypes differentially shape TME, suggesting that different factors influence the immune system machinery. Indeed, the mesenchymal subtype shows an increased immune infiltration and a worse prognosis as compared to the neural one [120]. One explanation lies on the correlation with IDH mutation status, especially in neural subtypes and low-grade gliomas, which determines hypermethylation of genome regions, including those involved in the transcription immune-response factors, such as human leukocyte antigen, decreasing MHC-I-mediated antigen presentation [121]. This phenomenon suggests that the modulation of the immune system represents one of the main hallmarks of GBM, allowing to communicate with other players and to induce other hallmarks. Multiple strategies are adopted by GBM to induce immune-response suppression, such as the secretion of immunosuppressive factors, TGF-β derived from GBM cells, microglia and TAMs, IL-10, prostaglandin E-2 and immune checkpoint molecules such as programmed death-1 (PD1), stimulating the formation of Treg cells or suppressing cytotoxic T cells and DCs, impeding an antitumoral immune response [122,123]. Moreover, the Programmed Death-Ligand 1 (PD-L1), a key factor of immune evasion, interacts with H-Ras, also leading to EMT promoting invasion and dissemination [124]. The immune-checkpoint receptors expression, such as PD-L1 or CTLA-4, exerts a direct suppression of adaptive immunity [125]. Other immunomodulatory signals, including enzymes such as IDO1, are involved in T-cell suppression and proliferation by reducing tryptophan levels, thus inhibiting immune-cell function and preventing DCs activation [126]. Microglia and macrophages are the largest tumor-infiltrating cell population with relevant functions for the acquisition of immunosuppressive phenotype; in particular, M1-like to M2-like macrophage shift is one of the main mechanisms regulating immunomodulatory response. Particularly, the M2-like phenotype includes three distinct classes: M2a, M2b, and M2c, involved in repair, immunoregulatory and acquired-deactivating phenotypes, respectively [127]. It has been shown that expression of the M2-like phenotype by TAMs is mediated by the secretion of soluble factors released by GBM cells, such as granulocyte macrophage colony-stimulating factor (GM-CSF) and interleukins, such as IL-4, IL-10, and IL-13 [128]. Immunosuppressive TAMs have protumoral properties, such as promoting vascularization, invasion, proliferation and immunosuppression [129]; TGF-β and IL-10 released by M2-like macrophages and by GBM cells stimulate capillary formation and angiogenesis interacting with endothelial cells and macrophages via αvβ3 integrin expression and SRC-PI3K-YAP signaling [130]. In addition, the PDGFB–PDGFRβ pathway is also involved in promoting vascularization, thanks to pericyte recruitment and migration mediated by CRCR1 [131]. The enhancement of CD8+ T-cell-mediated antitumor immunity has been associated to NF-κB-signaling activation in M2-like macrophages [132]. Immunosuppressive TAMs support GBM-invasion-expressing MMP-2 and MMP-9, which are also involved in angiogenesis, apoptosis, and cell proliferation; these hallmarks are fostered by the production of EGF, VEGF, and TGF-β1 by TAMs, which induce EMT in GBM cells [125].

**Figure 7:**
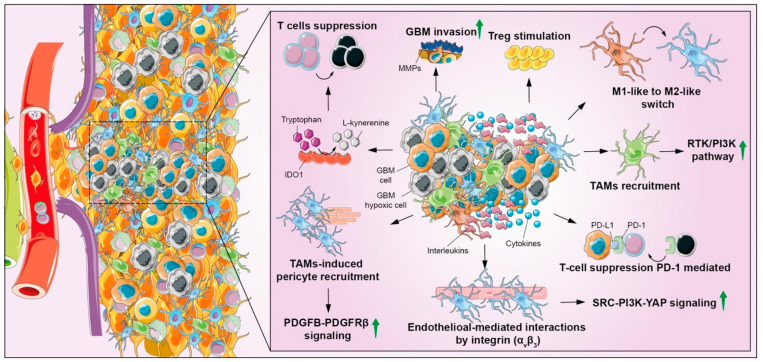
Representation of metabolic reprogramming processes connected GBM invasiveness. The support of GBM invasiveness in hypoxic conditions is not only directly linked to lactate production, but also by indirect effects such as the production of additional signals of molecules such as phosphoglucose isomerase/autocrine-motility factor (PGI/AMF), lipid metabolism and amino-acid reprogramming offer an important contribution to the proinvasion phenotype. Microglia adopts aerobic glycolysis by increasing lactate production and promoting M2 polarization with the concomitant production of cytokines and other factors that can directly suppress effector cells or indirectly via other types of immune cells such as intratumoral DCs and Treg cells, resulting in immunosuppression.

Hypoxia is considered among the main source of stimuli contributing to an immunosuppressive TME in GBM; the ECM protein periostin is highly expressed in hypoxic conditions, promoting the recruitment of TAMs, shifting towards an M2-like phenotype through the induction of RTK/PI3K pathway (Figure 7) [133]. However, GBM cells rely on stress-response mechanisms to maintain its influence on immune system; macroautophagy contributes to maintain a suppressive phenotype in both innate and adaptive immune cells; monocytes switch into immunosuppressive M2-like macrophages by autophagic signaling in response to colony-stimulating factor 1 (CSF1), whereas macroautophagy favors tumor tolerance by stimulation of FoxP3 T-regulatory-cell function as an adaptive immune response [134]. PD1 antibodies may be a promising strategy to overcome immune escape, but (CAR) T cells, oncolytic viruses, and vaccines are being developed to enhance the anticancer immune response. Checkpoint molecules are also valuable approaches to overcome adaptive resistance, but highly suppressive TMEs remain the main barrier. Therefore, additional therapies are tested including oncolytic virus and neoadjuvant anti-PD1 treatment. Further efforts to elucidate molecular and immunologic targets are required to increase therapeutic effects of such an approach [135].

## 8. Interconnections and Concluding Remarks

In recent years, several efforts have been made to expand and clarify the vision of GBM hallmarks. Dunn G.P. et al. reviewed cellular and molecular characteristics of GBM, focusing the role of RTKs in tumor progression and describing the invasion and angiogenesis as key tumor biological hallmarks [136]. Aum D.J. et al., addressed the GBM hallmarks by focusing on molecular heterogeneity and describing the classification of molecular subtypes with a particular consideration for GSCs [137]. A specific description of each individual hallmark discussing the biological mechanisms of GBM on replication, angiogenesis, reprogramming cellular energetics and immune evasion, has been reported [138]. Degl’Innocenti et al. elucidated how the future perspectives of integrated omics sciences, especially single-cell omics investigations, may contribute to exemplify genetic hallmarks of GBM [20], and recently, two review articles proposed how high-resolution in vivo imaging-technology approaches may support the understanding of GBM heterogeneity by the detection of specific hallmarks [139,140]. Nevertheless, a comprehensive and detailed view of the GBM hallmarks is needed and an explanation of the hallmarks by their mutual connections is still elusive. Interpretating the interconnected hallmarks of GBM is complex and deeply related to molecular interactions among them. The ultimate result would be to provide redundant information, as one hallmark is causing or is the result of another and it is often difficult to understand the cause–effect link. An example of such an intricate scenario is the vascularization and hypoxia circular positive loop. On one hand, tumor bulk growth induces sites not sufficiently perfused by the blood vessels, leading to necrosis but also to hypoxic niches; on the other hand, in hypoxic conditions, the expression of HIF orchestrates the complex transcriptional HRE response, determining the secretion of proangiogenic factors, such as VEGF, stimulating vascularization. Poorly organized vascular structures do not allow appropriate oxygen supply into the tumor, and here again the process begins.

The upregulation of PI3K/Akt/mTOR activation is another example of shared machinery among hallmarks, participating also in metabolic modulation by the regulation of glucose transporters, such as GLUT-1, and glycolytic enzymes such as HK2 determining a metabolic rewiring [141]. Overexpression of EGFR and TGF-β, regulated by HIF-1α/HIF-1β, promotes proliferation, differentiation, apoptosis, angiogenesis and invasion [142]. The role of hypoxia and invasion in GBM has been largely explored in literature, and this correlation was attributed to ECM degradation by many factors: (i) acidification of the extracellular milieu; (ii) upregulation of carbonic anhydrase; (iii) EGFRvIII/Integrins/FAK/SRC signaling; and iv) collagen crosslinking by procollagen lysine 2-oxoglutarate 5-dioxygenase upregulation [91,92]. In addition to ECM remodeling, the promotion of the EMT process was also recognized as a key contributor for invasiveness in hypoxic conditions; during EMT, the Notch pathway and TWIST1-induced signaling were induced and associated to cytoskeleton reorganization and motility by cyclin G2 upregulation [92]. GBM-cell migration toward blood vessels has been associated with stromal-derived factor 1 (SDF-1) upregulation and CXCR4 activation in hypoxic conditions [92]. CXCR4 is also involved in immunosuppressive mechanisms and microglia-induced GBM stimulation via PD1 [143,144]. Moreover, SDF-1 secreted by GSCs located in hypoxic niches promotes chemoattraction and activation of bone-marrow-derived endothelial progenitor cells that support endothelial survival and proliferation [145]. Immunomodulation correlates with HIF stabilization, since immunosuppressive M2-polarized TAMs, MDSCs, and Treg cells are recruited by GSCs in hypoxic niches [145].

It is possible to formulate the hypothesis that in GBM, as for many of cancer types, tumorigenesis can be described as development of sequence of events that leads to the disease with primary effects that trigger the tumorigenic cascades. However, the chronological sequence of such pathological events is still under investigation. Despite a certain number of triggering factors have been described, such as mutations linked to oncogenes and tumor suppressors for uncontrolled growth, identifying which one is the origin of the other is even more complex due to the creation of a TME influencing all cell populations within the tumor. Hence, the first event is the consideration that undifferentiated and potential staminal cells, such as GSCs, are critical players starting the cascade of uncontrolled growth and acquired anaplasia, pleomorphism, abnormal nuclear morphology, and loss of cell polarity. Then, cell-cycle deregulation generates an abnormal mass of tissue with unbalanced cell density supported by genetic alterations, which gives rise to an autonomous way to live without external stimuli, usually as result of oncogene activation and a clonal expansion of cells with stem-like properties. At this point, during the tumor growth, GBM-proliferating cells generate hypoxic cores surrounded by pseudo-palisade cells, rapidly dividing endothelial cells, pericytes, and smooth-muscle cells that form microvascular hyperplasia. The new vessels deliver oxygen and nutrient to the tumor cells and accelerate cancer-cell dissemination via an abnormal vascularization. The abnormal leaky vessels as well as vascular glomerular structures, in which endothelial cells and pericytes form poorly organized vascular structures, generate additional hypoxic niches, nearly to necrotic areas, surrounded by a row of hypoxic palisading tumor cells with lymphocytes, TAMs, and GSCs. The interaction between cancer cells, stroma, and immune cells became strong, influencing the composition and structure of the TME. Within the pleiotropic effects triggered by HIF and angiogenesis, invasion and migration are the most representative processes. It is worth noticing that for highly aggressive tumors, such as GBM, the definition of malignant tumors as a cellular mass able to invade and destroy adjacent structures and spread to distant sites with metastasis to cause death is not appropriate, since GBM does not have the fundamental characteristic of forming metastases. However, as mentioned above, the invasiveness of GBM is fully manifested with the involvement of other factors. For GBM, it is more appropriate that the typical condition of malignant tumors regarding the close correlation between stroma and parenchyma is strongly presented. Indeed, growth and progression of GBM cells are significantly dependent on the stromal component, which provides the essential structural framework for the tumor progression with a crosstalk between GBM and stromal cells that directly influences the tumor growth. Metabolic reprogramming and immunomodulation mechanisms will complete the final picture of such a malignant and aggressive tumor.

A definitive cure for GBM can reasonably be obtained by addressing three key issues: (1) deep knowledge of the pathophysiological mechanisms to detect prognostic and predictive biomarkers; (2) design of molecularly targeting drugs; (3) use of suitable therapeutical strategies (i.e., chemotherapy or radiation therapy) able to overcome drug and radioresistance, avoiding side effects and relapses. The first point may be placed at the top of a chain of events that could solve the other two; to date, identifying prognostic and predictive biomarkers seems to have placed the foundations for personalized treatments, as demonstrated by the application of TMZ depending on the IDH and MGMT methylation phenotype status. Certainly, current approaches are not sufficient to ensure remission from the disease so far, although they contribute to increased life expectancy. Targeted therapies have to face the obstacle of tumor heterogeneity and the interconnection between pathogenetic mechanisms of GBM that still remain far from being fully elucidated. In this regard, the combination of innovative drugs and radiotherapy treatments in view of the technological improvement linked to precision therapies such as hadron therapy or other sharpened forms of ionizing radiation can contribute to increased life expectancy of patients. Finally, it is necessary to focus studies towards the mechanisms of radio and drug resistance, which cover an important section of failure remission from this terrible disease. The integrated omics and molecular approaches will play an increasingly important role for the stratification of patients and for the understanding of undiscovered pathophysiological mechanisms.

## Data Availability

Not applicable.
